# Metabolic flux responses to genetic modification for shikimic acid production by *Bacillus subtilis* strains

**DOI:** 10.1186/1475-2859-13-40

**Published:** 2014-03-14

**Authors:** Dong-Feng Liu, Guo-Min Ai, Qing-Xiang Zheng, Chang Liu, Cheng-Ying Jiang, Li-Xia Liu, Bo Zhang, Yi-Ming Liu, Chen Yang, Shuang-Jiang Liu

**Affiliations:** 1 State Key Laboratory of Microbial Resources, Institute of Microbiology, Chinese Academy of Sciences, Beijing 100101, China; 2 School of Life Sciences, University of Science and Technology of China, Hefei 230026, China; 3 Key Laboratory of Synthetic Biology, Institute of Plant Physiology and Ecology, Shanghai Institute for Biological Sciences, Chinese Academy of Sciences, Shanghai 200032, China; 4 Institute of Microbiology, Chinese Academy of Sciences, Beichen-Xilu, Beijing, Chaoyang District 100101, China

**Keywords:** Shikimic acid production, Shikimate pathway, *Bacillus subtilis*, Metabolic flux assay (MFA), *aroA*, *aroD*, *tkt*, *pyk*

## Abstract

**Background:**

Shikimic acid (SA) is a key chiral starting molecule for the synthesis of the neuramidase inhibitor GS4104 against viral influenza. Microbial production of SA has been extensively investigated in *Escherichia coli*, and to a less extent in *Bacillus subtilis*. However, metabolic flux of the high SA-producing strains has not been explored. In this study, we constructed with genetic manipulation and further determined metabolic flux with ^13^C-labeling test of high SA-producing *B. subtilis* strains.

**Results:**

*B. subtilis* 1A474 had a mutation in SA kinase gene (*aroI*) and accumulated 1.5 g/L of SA. Overexpression of plasmid-encoded *aroA*, *aroB*, *aroC* or *aroD* in *B. subtilis* revealed that *aroD* had the most significantly positive effects on SA production. Simultaneous overexpression of genes for 3-deoxy-D-arabinoheptulosonate-7-phosphate synthase (*aroA*) and SA dehydrogenase (*aroD*) in *B. subtilis* BSSA/pSAAroA/pDGSA*AroD* resulted in SA production of 3.2 g/L. ^13^C-Metabolic flux assay (MFA) on the two strains BSSA/pHCMC04/pDG148-stu and BSSA/pSA*AroA*/pDGSA*AroD* indicated the carbon flux from glucose to SA increased to 4.6% in BSSA/pSA*AroA*/pDGSA*AroD* from 1.9% in strain BSSA/pHCMC04/pDG148-stu. The carbon flux through tricarboxylic acid cycle significantly reduced, while responses of the pentose phosphate pathway and the glycolysis to high SA production were rather weak, in the strain BSSA/pSA*AroA*/pDGSA*AroD*. Based on the results from MFA, two potential targets for further optimization of SA production were identified. Experiments on genetic deletion of phosphoenoylpyruvate kinase gene confirmed its positive influence on SA production, while the overexpression of the transketolase gene did not lead to increase in SA production.

**Conclusion:**

Of the genes involved in shikimate pathway in *B. subtilis*, *aroD* exerted most significant influence on SA accumulation. Overexpression of plasmid-encoded *aroA* and *aroD* doubled SA production than its parent strain. MFA revealed metabolic flux redistribution among phosphate pentose pathway, glycolysis, TCA cycle in the low and high SA-producing *B. subtilis* strains. The high SA producing strain BSSA/pSA*AroA*/pDGSA*AroD* had increased carbon flux into shikimate pathway and reduced flux into TCA cycle.

## Background

Shikimic acid (3,4,5-trihydroxy-1-cyclohexene-1-carboxylic acid, SA), is a key chiral starting material for the synthesis of the antiviral neuraminidase inhibitor GS4104 [1,2]. It is a key metabolic intermediate of the shikimate pathway for biosynthesis of aromatic amino acids (L-Phe, L-Trp, and L-Tyr) and many alkaloids in plants and microorganisms [3-5]. As a commercial product, SA has been extracted from the fruits of the *Illicium* plant. However, microbial fermentation as an alternative process for SA production has attracted more and more interests.

**Figure 1:**
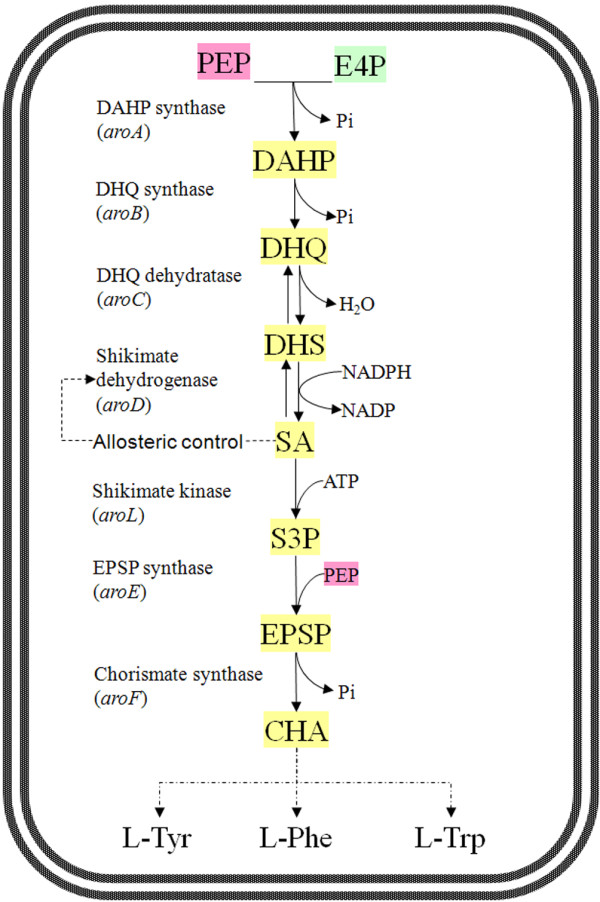
**Pathway of shikimic acid biosynthesis and its regulation in *B. subtilis*.** Abbreviations used: CHA, chorismate; DAHP, 3-deoxy-d-arobino-heptulosonate 7-phosphate; DHQ, 3-dehydroquinate; DHS, 3-dehydroshikimate; EPSP, 5-enolpyruvoylshikimate 3-phosphate; E4P, erythrose 4-phosphate; L-Phe, L-phenylalanine; L-Trp, L-tryptophan; L-Tyr, L-tyrosine; PEP, phosphoenolpyruvate; SA, shikimate; S3P, shikimate 3-phosphate.

Beginning with phosphoenolpyruvate (PEP) and erythrose-4-phosphate (E4P), SA is synthesized via first 4 reactions of the shikimate pathway (Figure 1). This shikimate pathway is essential to bacterial growth and there has been no report on excessive accumulation of SA by microorganisms. Genetic manipulation and metabolic engineering approaches had been applied to modify several microorganisms, particular *Escherichia coli*, for SA accumulation [6-9]. One strategy was to increase the supply of PEP and/or E4P, thus more carbon substrate channeled into SA synthesis. For examples, the PEP-consuming glucose transfer system (PTS) was replaced by non-PEP-consuming glucose facilitators, and transketolase I (*tktA*) and PEP synthase (*ppsA*) genes were overexpressed to increase the availability of E4P and PEP [10-14]. A second strategy was to block SA flux into chorismic acid by inactivating SA kinases (*aroK* and a*roL*) genes, and resulting the accumulation of SA in large quantity [6,15]. A third strategy was overexpression of feedback-resistant (fbr) 3-deoxy-D-arabinoheptulosonate-7-phosphate (DAHP) synthase genes (e.g. *aroF*^*fbr*^ and *aroG*^*fbr*^), thus avoided feedback inhibition in the first step of the shikimate pathway [7,16]. It was reported that high amount of extracellular shikimic acids activated the shikimic acid transporter ShiA (*shiA*) and invoked transport of SA back into cells. So, a forth strategy was to inactivate the *shiA* thus vanished the uptake of SA back into cell and diminished intracellular accumulation of 3-dehydroshikimic acid as well [9]. Besides *E. coli*, efforts were also made to convert *Bacillus subtilis* into a SA accumulator. According to a patent description [17], genetic deletion of SA kinase gene (*aroI*) and overexpression of bacilli DAHP synthase (*aroA*) and SA dehydrogenase (*aroD*) genes resulted in a significant accumulation of SA in *B. subtilis*. So far as we know, intensive evaluation of *aro* genes on SA production has not been reported in *B. subtilis*. Furthermore, knowledge of the metabolic fluxes of those genetically engineered strains of *E. coli* and *B. subtilis* is still missing.

In this study, we investigated the metabolic fluxes with ^13^C-labeled glucose in two genetically modified, SA-producing strains of *B. subtilis*, namely BSSA/pHCMC04/pDG148-stu and BSSA/pSA*AroA*/pDGSA*AroD*. Our results indicated that significant metabolic flux changes occurred for the shikimate pathway, tricarboxylic acid (TCA) cycle, and reactions involving PEP and E4P generation and consumption. Based on the ^13^C-labeling metabolic flux assay (MFA), we further created mutants for SA production. The highest SA-producing *B. subtilis* strain produced 3.46 g/L of SA during batch cultivation in flasks.

## Results

### Strain construction and SA productivity

*B. subtilis* strain 1A474 had been used for genome mapping and its genome had a mutation on the SA kinase gene (*aroI*). In this study, we identified that this specific mutation of *aroI* was resulted from a single mutation of C to G, leading to Arg^129^ (coded by CGC) being replaced by Cys^129^ (UGC). Phenotypically, strain 1A474 was not able to grow on minimal medium and it restored growth when supplemented with L-Phe, L-Trp, and L-Tyr. We determined that strain 1A474 accumulated up to 1.5 g/L of SA in batch cultivation (Table 1).

**Table 1:** **Production of shikimic acid by various recombinant strains of *Bacillus subtilis***

**Strains**	Shikimic acid*(g/L)	**Changes (%)**	**Notes**^**#**^
1A474	1.50 ± 0.22	100.0	Parent strain
BSSA/pHCMC04	1.81 ± 0.18	120.7	Parent strain carrying pHCMC04
BSSA/pSA*AroA*	1.42 ± 0.20	94.7	Overexpression of *aroA*
BSSA/pSA*AroB*	1.74 ± 0.23	116.0	Overexpression of *aroB*
BSSA/pSA*AroC*	1.77 ± 0.15	118.0	Overexpression of *aroC*
BSSA/pSA*AroD*	2.30 ± 0.17	153.3	Overexpression of *aroD*
BSSA/pSA*AroA*/ pDGSA*AroD*	3.20 ± 0.07	213.3	Overexpression of *aroD* plus *aroA*
BSSA/pSA*AroB*/pDGSA*AroD*	2.99 ± 0.05	199.3	Overexpression of *aroD* plus *aroB*
BSSA/pSA*AroC*/pDGSA*AroD*	2.90 ± 0.03	199.3	Overexpression of *aroD* plus *aroC*
BSSA/pSA*AroD* pDGSA*AroD*	2.91 ± 0.04	194.0	Overexpression of *aro*D on two plasmids
BSSA(Ω*tkt*::pSATkt)	3.11 ± 0.03	207.3	Overexpression of *tkt* in BSSA/pSA*AroA*/pDGSA*AroD*
BSSA(Ω*pyk*::pSAPyk)	3.46 ± 0.04	230.7	Knockout of *pyk* in BSSA/pSA*AroA*/pDGSA*AroD*

*The shikimic acid concentrations in the culture supernatants were averages of the results from three parallel cultures and standard errors were provided for shikimic acid production.

^#^For detailed genotypes, see Table 3.

Strain 1A474 was used to construct high SA producers. For this purpose, we adopted a strategy to overexpress *aro* genes involving shikimate pathway in strain 1A474. The gene *aroA*, *aroB*, *aroC*, and *aroD* were cloned from the genome of strain 168 and were individually overexpressed in strain 1A474. SA production by the resulting recombinant strains was determined and was compared to their parent strains. Results showed that overexpression of plasmid-encoded *aroA* (strain BSSA/pSA*AroA*), *aroB* (strain BSSA/pSA*AroB*), or *aroC* (strain BSSA/pSA*AroC*), exhibited no significant increase in SA production, however, the overexpression of plasmid-encoded *aroD* in strain BSSA/pSA*AroD* resulted in higher production of SA (2.3 g/L, an increase of 53%), compared to its parent strain (1.5 g/L) (Table 1).

In order to improve SA production further, we co-overexpressed *aroA*, *aroB*, or *aroC* with *aroD* in *B. subtilis*. Results showed that co-overexpression of *aroA* (strain BSSA/pSA*AroA*/pDGSA*AroD*), *aroB* (strain BSSA/pSA*AroB*/pDGSA*AroD*), or *aroC* (strain BSSA/pSA*AroC*/pDGSA*AroD*) with *aroD* indeed increased SA production compared to strain BSSA/pHCMC04/ pDGSA*AroD* and parent strain 1A474 (Table 1). We also found that double overexpression of *aroD* on two plasmids further increased SA production compared to single overexpression of *aroD* (Table 1). Strain BSSA/pSA*AroA*/pDGSA*AroD* that co-overexpressed *aroA* and *aroD* was the best SA producer, with SA production of 3.2 g/L during batch cultivation fermentation. Compared to the parent strain 1A474, SA production was doubled in strain BSSA/pSA*AroA*/pDGSA*AroD* (Table 1).

### Phenotypic characterization of growth, glucose consumption and metabolite production with strains BSSA474a and BSSA47407

**Figure 2:**
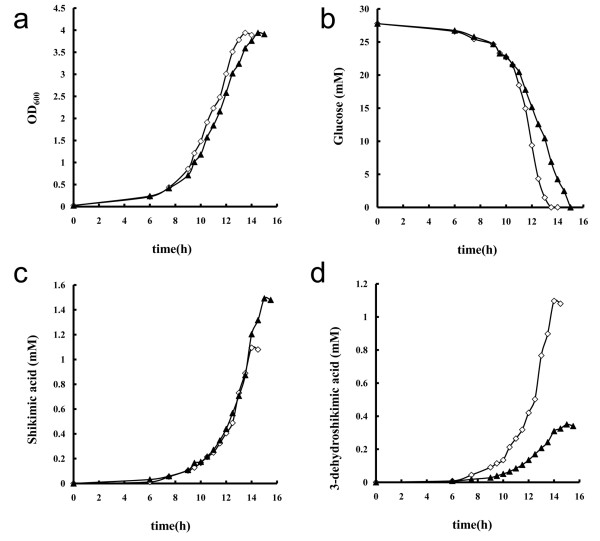
**Time courses of cell growth (a), glucose consumption (b), shikimic acid production (c), and 3-dehydroshikimic acid production (d) of *B. subtilis *** **BSSA/pHCMC04/pDG148-stu (◊) and BSSA/pSA** ***AroA*/pDGSA** ***AroD *** **(▲) in M9 minimal medium supplemented with 5 g/L glucose.**

Figure 2 showed cell growth (OD_600_), glucose consumption, SA production, and 3-dehydroshikimic acid production of strains BSSA/pHCMC04/pDG148-stu and BSSA/pSA*AroA*/pDGSA*AroD* in M9 minimal medium supplemented with 5 g/L glucose. Strain BSSA/pHCMC04/pDG148-stu grew slightly faster than BSSA/pSA*AroA*/pDGSA*AroD* did, but they reached almost identical cell mass (OD_600_) at the end of cultivation. The consumption of glucose was faster by BSSA/pHCMC04/pDG148-stu than BSSA/pSA*AroA*/pDGSA*AroD*. The concentrations of two metabolites, namely SA and 3-dehydroshikimic acid, were determined. Strain BSSA/pHCMC04/pDG148-stu produced equal amounts of SA and 3-dehydroshikimic acid (Table 2, 1.1 mM), at a molar ratio of 1:1. Strain BSSA/pSA*AroA*/pDGSA*AroD* accumulated more SA (1.49 mM) than 3-dehydroshikimic acid (0.35 mM), at a molar ratio of 4.3:1. For the purpose of metabolic flux analysis, we calculated the specific cell growth (μ), specific glucose consumption (q_glu_), specific shikimic acid production (q_sa_), and specific 3-dehydroshikimic acid production (q_dhs_) rates, and they are listed in Table 2.

**Table 2:** **Determination of growth (OD**_**600**_**), specific cell growth rate (μ), specific glucose consumption rate (q**_**glu**_**), specific shikimic acid production rate (q**_**sa**_**), and specific 3-dehydroshikimic acid production rate (q**_**dhs**_**) of *B. subtilis *strains BSSA/pHCMC04/ pDG148-stu and** BSSA/pSA*AroA*/pDGSA*AroD*

**Strains**	**μ(h**^**-1**^**)**	**q**_**glu**_**(mmol g**^**-1**^ **h**^**-1**^**)**	**q**_**sa**_**(mmol g**^**-1**^ **h**^**-1**^**)**	**q**_**dhs**_**(mmol g**^**-1**^ **h**^**-1**^**)**
BSSA/pHCMC04/ pDG148-stu	0.434 ± 0.023	5.199 ± 0.120	0.101 ± 0.011	0.132 ± 0.013
BSSA/pSA*AroA*/pDGSA*AroD*	0.332 ± 0.015	4.666 ± 0.132	0.159 ± 0.014	0.048 ± 0.007

Data are from 3 parallel cultivations and standard deviations are provided. *B. subtilis* strains were cultivaeted 250-mL flasks that contained 50 mL of M9 minimal medium, which was supplemented to 5 g/L glucose. Temperature and the shaking rate of the incubator were held constantly at 37°C and 200 rpm, respectively.

### Metabolic flux responses to high production of SA in strain BSSA47407

#### Metabolic flux analysis by GC/LC-MS

**Figure 3:**
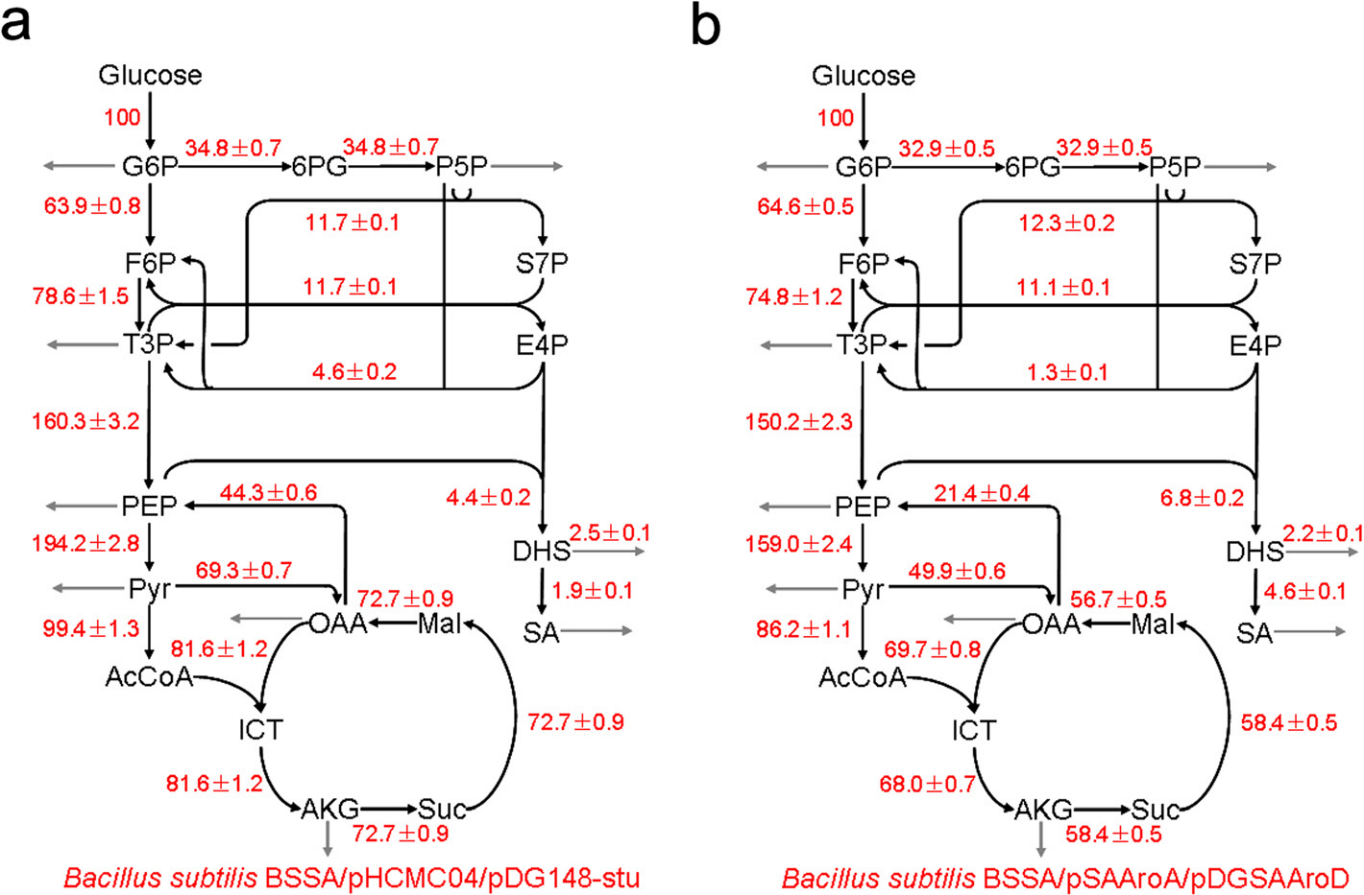
**Metabolic flux distribution in *B. subtilis *BSSA/pHCMC04/pDG148-stu (a) and BSSA/pSA*AroA*/pDGSA*AroD *(b) during exponential growth phase.** The estimated net fluxes were percentages of the relative rates normalized to the glucose uptake rates. Directions of net fluxes were represented by arrows. The gray arrows indicated flux related to biomass formation and fermentation products. The flux distributions were obtained from the best fit to the quantitative physiological data and the constraints derived from the MS measurements. F6P, fructose-6-phosphate; GAP, Glyceraldehyde 3-phosphate; AcCoA, acetyl-CoA; ICT, isocitrate; AKG, a-ketoglutarate; S7P, sedoheptulose-7-phosphate; DHS, 3-dehydroshikimic acid; SA, shikimic acid.

The ^13^C-based metabolic flux analysis was performed for the strains BSSA/pHCMC04/pDG148-stu and BSSA/pSA*AroA*/pDGSA*AroD* grown in the medium containing either 100% [1-^13^C] glucose or 20% [U-^13^C] glucose and 80% unlabeled glucose. The mass isotopomer patterns in cellular amino acids were analyzed by GC-MS (Additional file 1: Table S1). The intracellular phenylalanine and tyrosine were not labeled by ^13^C because the SA kinase gene (*aroI*) was mutated in both strains and *de nova* synthesis of these amino acids from glucose was blocked. We measured the ^13^C label state of extracellular shikimate by using LC-MS (Additional file 1: Table S1). To assess if the isotopic steady state was achieved, samples were taken at different time points during the exponential growth phase. The determined mass isotopomer distributions of key amino acids were almost unchanged with the time of harvest, which showed that a (quasi-) steady state could be reached during the exponential growth phase in batch cultures. From the GC-MS and LC-MS data (Additional file 1: Table S1), the ^13^C labeling patterns of the precursor metabolites were identified, which allowed us to determine the metabolic flux ratios. The absolute net fluxes were then quantified by combining the flux ratios, physiological data (Table 1), and the biomass composition data [18]. The intracellular flux distribution in the parent strain BSSA/pHCMC04/pDG148-stu and high SA-producing strain BSSA/pSA*AroA*/pDGSA*AroD* are shown in Figure 3.

#### Overall metabolic fluxes and responses of shikimate pathway

We observed a significant flux redistribution of several metabolic reactions in responses to the overexpression of plasmid-encoded *aro* genes in the high SA-producing strain BSSA/pSA*AroA*/pDGSA*AroD*. The overall conversion of glucose into shikimate pathway increased to 6.8% in strain BSSA/pSA*AroA*/pDGSA*AroD* from 4.4% in strain BSSA/pHCMC04/pDG148-stu. Consequently, the metabolic flux to SA increased from 1.9% in strain BSSA/pHCMC04/pDG148-stu to 4.6% in strain BSSA/pSA*AroA*/pDGSA*AroD*. We observed that the production rate of 3-dehydroshikimic acid decreased to 0.048 mmol g^-1^ h^-1^ in strain BSSA/pSA*AroA*/pDGSA*AroD* from 0.132 mmol g^-1^ h^-1^ in the parent strain BSSA/pHCMC04/pDG148-stu (Table 2). Correspondingly, the metabolic flux from E4P and PEP into DHS decreased from 2.5% in the parent strain BSSA/pSA*AroA*/pDGSA*AroD* to 2.2% in strain BSSA/pSA*AroA*/pDGSA*AroD*. The reversible reaction converting DHS to SA and being catalyzed by SA dehydrogenase (AroD) increased its flux by 2.4 folds (from 1.9% in BSSA/pHCMC04/pDG148-stu to 4.6% in BSSA/pSA*AroA*/pDGSA*AroD*) in strain BSSA47407 (Figure 3).

#### Flux shifts of TCA cycle, EMP and PPP pathways

As shown in Figure 3, the flux through the TCA cycle was 81.6% relative to the glucose uptake rate in BSSA/pHCMC04/pDG148-stu, while this flux decreased to 69.7% in BSSA/pSA*AroA*/pDGSA*AroD*. The overall flux of TCA cycle reduced significantly in the high SA-producing strain BSSA/pSA*AroA*/pDGSA*AroD*, suggesting a weak TCA cycle during high production of SA with *B. subtilis*. In contrast, fluxes of the EMP and pentose phosphate pathways did not shift significantly, except the reactions involving E4P generation and consumption. In the PPP pathway, transketolase, encoded by *tkt*, catalyzes reversible reaction (see Additional file 1: Appendix S1) and is that to E4P flux. We did not observe significant changes of flux driven by the transaldolase (*v*_6_). However, the net flux of reaction encoded by *tkt* decreased from 4.6% in the parent strain BSSA/pHCMC04/pDG148-stu to 1.3% in the high SA-producing strain BSSA/pSA*AroA*/pDGSA*AroD*, suggesting that this reaction was severely disturbed and might be interesting to see how the SA synthesis would be influenced if *tkt* were overexpressed.

#### Supply of PEP for SA synthesis

PEP is one of the initial substrates for shikimate synthesis. In almost all known microorganisms, the DAHP synthase catalyzes the condensation of PEP and E4P, which is the first reaction of the shikimate pathway. It is reasonable to consider any changes of PEP generation and consumption in the high SA-producing BSSA/pSA*AroA*/pDGSA*AroD*. Two reactions were considered in this study for generation of PEP: 1) The enolase (NP_391270.1, EC:4.2.1.11) catalyzes glycerate-2-phosphate into PEP (*v*_8_, see Additional file 1: Appendix S1). 2) The PEP-forming ATP:oxaloacetate carboxylyase (also named PEP carboxykinase, PckA, NP_390934.2, EC:4.1.1.49) catalyzes the conversion of oxaloacetate into PEP (*v*_10_, see Additional file 1: Appendix S1). As shown in Figure 3, flux into reaction *v*_8_ decreased from 160.3% in BSSA/pHCMC04/pDG148-stu to 150.2% in BSSA/pSA*AroA*/pDGSA*AroD*, and flux into reaction *v*_10_ reduced from 44.3% in BSSA/pHCMC04/pDG148-stu to 21.4% in BSSA/pSA*AroA*/pDGSA*AroD*. Meanwhile, we also observed that the conversion of PEP into pyruvate, which is encoded by *pyk*, also decreased, from 194.2% in BSSA/pHCMC04/pDG148-stu to 159.0% in BSSA/pSA*AroA*/pDGSA*AroD*. Taking all these flux information, we considered that the supply of PEP might in shortage in the high SA-producing BSSA/pSA*AroA*/pDGSA*AroD*. In addition, we observed that the flux of reaction (*v*_9_) in the SA-producing BSSA/pSA*AroA*/pDGSA*AroD* was still high (159%). For the purpose to increase supply of PEP to shikimate synthesis, knockout of this *pyk* might be a choice to increase SA production.

### Validation of MFA results: effects of *tkt* overexpression and *pyk* deletion on SA production

Based on the previous results, we selected two targets for further genetic manipulation and to improve the SA production in *B. subtilis*. For the purpose to increase carbon flux to PEP, efforts were made to overexpress *tkt* in BSSA/pSA*AroA*/pDGSA*AroD*. However, the resulting strain BSSA/Ω*tkt*::pSA*tkt* did not show significant changes in SA production (Table 1). Further efforts were make to knock out the pyruvate kinase thus would diminish the flux of PEP into pyruvate. The resulting strain BSSA/Ω*pyk*::pSA*pyk* produced 3.46 g/L of SA, an increase of 8% compared to its parent strain BSSA/pSA*AroA*/pDGSA*AroD*.

## Discussion

In this study, we investigated the influence of overexpression of *aro* genes on the accumulation of SA, and we constructed high SA producers by overexpression of *aroA* and *aroD* in *B. subtilis*. Our results demonstrated that overexpression of plasmid-encoded *aroD* increased the accumulation of SA, suggesting that AroD-driven reduction of 3-dehydroshikimate into SA was rate-limiting step for SA accumulation in *B. subtilis*. This is different from that of *E. coli*, of which the condensation of PEP and E4P into DAHP is the rate-limiting reaction and this reaction is subjected to extensive feedback regulations [19-21].

^13^C-MFA revealed carbon flux redistributions into shikimate pathway and TCA cycle, and reactions involving generation and consumption of PEP and E4P in genetically modified *B. subtilis* for SA production. Based on the MFA results in this study and previous investigation on *E. coli*[7,8,13], *tkt* and *pyk* were selected to be targets for genetically modification and for improvement of SA production with *B. subtilis*. Previous investigations showed that transformation with plasmid carrying *tktA* that encodes transketolase I increased the availability of E4P, and therefore increased SA and aromatic amino acid productions in *E. coli*[13]*.* However, this study showed that overexpression of *tkt* did not increased SA production in *B. subtilis*, a remarkable difference from that in *E. coli*. Since transketolase catalyzes a reversible reaction (see Additional file 1: Appendix S1), i.e., it generates E4P in one direction but consumes E4P in the other direction, we deduced that the *tkt*-encoded transketolase in *B. subtilis* catalyzed reaction more favored to direction of fructose 6-phosphate and glyceraldehyde 3-phosphate, and that the *tktI*-encoded transketolase in *E. coli* more favored the formation of E4P and xylulose 5-phosphate. Confirmation of this deduction needs further biochemical evidences on catalytic properties of the transketolases from *B. subtilis* and *E. coli*. These results suggest that MFA data need to be carefully interpreted and conclusions from MFA need to be experimentally confirmed, and application of MFA data from one bacterial species may be different from that of another bacterial species.

Based on MFA results as well as previous investigations in *E. coli*[7], a second target was spotted for knockout of *pyk*, so that consumption of PEP by this Pyk-driven reaction would be vanished. Pyruvate kinase, encoded by *pyk*, is an enzyme involved in glycolysis and catalyzes the consumption of one molecule of PEP, yielding one molecule of pyruvate and one molecule of ATP. Previous investigation revealed that PEP increased its intracellular pools when *pyk* was deleted [22]. We observed that SA production increased by 8% in *B. subtilis*, from 3.2 g/L in the control strain BSSA/pSA*AroA*/pDGSA*AroD* to 3.46 g/L in strain BSSA/Ω*pyk*::pSA*pyk*. This result is again different from that observed in *E. coli pyk* mutant, which did not showed obvious increase of intracellular PEP level [22]. Careful exploration of metabolic networks in *B. subtilis* and *E. coli* suggested that they have very different reactions at the PEP-pyruvate-oxaloacetate node, particularly the reactions for PEP and oxaloacetate generation/consumption [23]. As demonstrated in previous studies, the *pyk* mutant of *B. subtilis* maintained high intracellular PEP levels [22] and produced less acetic acid than wild type did. This invoked the enthusiasm on exploitation of *B. subtilis pyk* mutants for production of various aromatic compounds. For example, *B. subtilis* strain BSTZ0402, an inducible *pyk* mutant, had been successfully constructed and yielded three folds higher folic acids compared to the parent strain *B. subtilis* 168 [24]. Additional works on mining the ^13^C-MFA data and exploitation of metabolic network from *B. subtilis* for improving SA productivity are in progress.

## Conclusion

Based on the data obtained in this study, it was concluded that overexpression of plasmid-encoded *aroD* exerted most significant influence on SA accumulation in *B. subtilis*. Co-everexpression of plasmid-encoded *aroA* and *aroD* doubled SA production than its parent strain BSSA/pHCMC04. So far as we know, this is the first time to quantify the flux flow in SA producing *B. subtilis* strains. MFA revealed metabolic flux redistribution among phosphate pentose pathway, glycolysis, TCA cycle in the low and high SA-producing *B. subtilis* strains. The high SA producing strain BSSA/pSA*AroA*/pDGSA*AroD* had increased carbon flux into shikimate pathway and reduced flux into TCA cycle. Based on the MFA results, *tkt* and *pyk* were targeted for improving SA production by *B. subtilis*. Overexpression of plasmid-encoded *tkt* did not increase SA production, and the deletion of *pyk* increased SA production by 8% in *B. subtilis*. The results showed that *B. subtilis* and *E. coli* had very different responses to genetic manipulations of the *aro* genes involving shikimate pathway and the *tkt* and *pyk* involving PEP/E4P generation and consumption.

## Materials and methods

### Microorganisms, plasmids, cultivation, and determination of cell growth

The bacterial strains, plasmids, and primers used in this study are listed in Table 3. *B. subtilis* strains 1A474 and 168 were obtained from the Bacillus Genetic Stock Center (BGSC) at the Ohio State University (http://www.bgsc.org/). Both *B. subtilis* and *E. coli* were routinely cultivated in Luria Bertani (LB) broth at 37°C. When needed, antibiotics were included at the following concentrations, 15 μg/mL of erythromycin, chloramphenicol of 5 μg/mL, ampicillin of 100 μg/mL and neomycin of 30 μg/mL.

**Table 3:** ***Bacillus subtilis*** **strains, plasmids, and primers used in this study**

**Strains/plasmids/primers**	**Relevant characteristics**	**Source/notes**
**Strains**		
168	*trpC2*	BGSC
1A474	*amyE3 aroI10*	BGSC
BSSA/pHCMC04	1A474 carrying pHCMC04	This work
BSSA/pSA*AroA*	1A474 carrying pSA*AroA*	This work
BSSA/pSA*AroB*	1A474 carrying pSA*AroB*	This work
BSSA/pSA*AroC*	1A474 carrying pSA*AroC*	This work
BSSA/pSA*AroD*	1A474 carrying pSA*AroD*	This work
BSSA/pHCMC04/ pDG148-stu	1A474 carrying pHCMC04 and pDG148-stu	This work
BSSA/pHCMC04/ pDGSA*AroD*	1A474 carrying pHCMC04 and pDGSA*AroD*	This work
BSSA/pSA*AroA*/ pDGSA*AroD*	1A474 carrying pSA*AroA* and pDGSA*AroD*	This work
BSSA/pSA*AroB* pDGSA*AroD*	1A474 carrying pSA*AroB* and pDGSA*AroD*	This work
BSSA/pSA*AroC* pDGSA*AroD*	1A474 carrying pSA*AroC* and pDGSA*AroD*	This work
BSSA/pSA*AroD* pDGSA*AroD*	1A474 carrying pSA*AroD* and pDGSA*AroD*	This work
BSSA(Ω*tkt*::pSATkt)	BSSA/pSA*AroA*/pDGSA*AroD* harboring Ω*tkt*::pSATKT	This work
BSSA(Ω*pyk*::pSAPyk)	BSSA/pSA*AroA*/pDGSA*AroD* harboring Ω*pyk*::pSAPYK	This work
**Plasmids**		
pHCMC04	*bla* P_*xyl*A_*xylR cat*	BGSC
pDG148-stu	*neo ble* P*spac lacI bla*	BGSC
pMUTIN4	*bla* P*spac lacZ lacI ermC*	BGSC
pSA*AroA*	pHCMC04 derivative, *aroA* expressed from P_*xyl*A_	This work
pSA*AroB*	pHCMC04 derivative, *aroB* expressed from P_*xyl*A_	This work
pSA*AroC*	pHCMC04 derivative, *aroC* expressed from P_*xyl*A_	This work
pSA*AroD*	pHCMC04 derivative, *aroD* expressed from P_*xyl*A_	This work
pDGS*AroD*	pDG148-stu derivative, *aroD* expressed from P*spac*	This work
pSATkt	*bla* P*spac*-*tkt*-*lacZ lacI ermC*	This work
pSAPyk	*bla* P*spac*-*pyk*’-*lacZ lacI ermC*	This work
**Primers**		
*aroA*-fw-*Spe*I	TCAACTAGTATGAGCAACACAGAGTTAG	Cloning of *aroA* into pHCMC04
*aroA*-rv-*Xma*I	TCACCCGGGTTAAGCGTTGACTTTCAC	
*aroB*-fw-*Spe*I	TCCACTAGTATGAAGACACTGCATGTTC	Cloning of *aroB* into pHCMC04
*aroB*-rv-*Xma*I	TCAGGATCCTCATGATGTCTCCTCCAATC	
*aroC*-fw-*Spe*I	TCAACTAGTGTGAACGTGTTAACGATTAAAG	Cloning of *aroC* into pHCMC04
*aroC*-rv-*Xma*I	TCACCCGGGCTATCCCCGTGTGTTTTTATG	
*aroD*-fw-*Spe*I	TCCACTAGTATGAAAAAGCTGTACGG	Cloningof *aroD* into pHCMC04
*aroD*-rv-*Xma*I	TCAGGATCCTTAACATTCTGTTCCTCC	
*aroD*-DG-fw	AAGGAGGAAGCAGGTATGAAAAAGCTGTACGG	Cloning of *aroD* into pDG148-stu
*aroD*-DG-rv	GACACGCACGAGGTTTAACATTCTGTTCCTCC	
*tkt*-Mu-fw-*Xma*I	ACTCCCGGGACATAAGGAAGGGGATTTTTATG	Cloning of *tkt* into pMUTIN4
*tkt*-Mu-rv-*Bam*HI	ACTGGATCCACTCATCCTCTTTCAAAAGC	
*tkt*-ck-fw	TTTCAGGAATACATAGAG	Confirming the integration of pSATKT
*tkt*-ck-rv	TTAGACAAAATTTCTTTC	
*pyk*-Mu-fw-*Xma*I	CCCCCGGGGAAGATTTCAGAAGGAAGTGAACC	Cloning 5’ end of *pyk* into pMUTIN4
*pyk*-Mu-rv-*Bam*HI	CCGGATCCGGAATTTCCACACCTAAGTCTCC	
*pyk*-ck-fw	AACGTACATATGTAATCG	Confirming the integration of pSAPYK
*pyk*-ck-rv	CCGTTCAGCAAAAATAAAAC	

BGSC: Bacillus Genetic Stock Center. Resistance gene abbreviations as follows: *bla*, ampicillin; *ermC*, erythromycin and lincomycin; *cat*, chloramphenicol; *neo*, kanamycin. Other abbreviation: Ω, insertion of integrated plasmid. Restriction sites were underlined.

Cell growth was monitored by optical density measurements at 600 nm (OD_600_) using a UV/visible spectrophotometer. Cellular dry weights were determined with duplicate samples of culture broth, by precipitation of cells and washed with distilled water and lyophilization.

### Determination of SA and 3-dehydroshikimic acid by high performance liquid chromatography (HPLC)

*B. subtilis* was cultivated in LB broth at 37°C and at 200 rpm, overnight, and cells from culture were used as inoculum. SA production was determined in triplicates after 90 h cultivation at 37°C and 200 rpm in 250-mL flasks that contained 50 mL of fermentation broth. The fermentation broth was modified from Iomantas et al. [17] by deletion of maltose, and had the following components (g/L): K_2_HPO_4_ · 3H_2_O (18.3); KH_2_PO_4_ (6); Urea (6); sucrose (80); MgSO_4_ · 7H_2_O (1); FeSO_4_ (0.01); MnSO_4_ (0.01); Yeast Extract (4). For induction of the target gene overexpression in recombinant *B. subtilis* strains, IPTG at final concentration of 0.05 mM and xylose at final concentration of 5 g/L were added into the fermentation broth.

SA and 3-dehydroskimic acid accumulation in culture supernatant was determined with an HPLC system (Agilent 1200 series) equipped with a SB C18 column (4.6 mm × 250 mm × 5 μm). Five ml samples of fermentation broth were centrifuged at 13,000 rpm for 5 min on Microfuge® 18 centrifuge (BECKMAN COULTER^TM^) to remove cells, and the supernatant was applied for HPLC analysis. The HPLC was run with mixture of solution A (phosphoric acid in water, pH 2.5) and solution B (methanol) as eluant and was operated at a flow rate of 0.35 mL/min. SA and 3-dehydroshikimic acid were detected at 215 nm with adiode array detector. The following gradient was used: at 0–7.5 min, 95% of solution A and 5% of solution B; at 7.5-8.0 min, 100% of solution B; at 8.0-15 min, 100% of solution B; 15.0-15.5 min, 95% of solution A and 5% of solution B; 15.5-22.5 min, 95% of solution A and 5% of solution B. Standard SA and 3-hydroskimic acid were eluted at 5.411 and 6.241 min, respectively, under these conditions.

### Determination of glucose concentrations

Glucose concentrations were enzymatically determined with glucose oxidase kit by using a bioanalyzer, according to producer’s description (SBA-40C, Shandong Academy of Sciences).

### DNA extraction, amplification, plasmid construction and genetic transformation

Plasmid and chromosomal DNAs were isolated using the Plasmid Minspin HP Kit and the SiMax™ Genome DNA Kit, respectively. Purification of DNA fragments from agarose gels was done with the TIANgel Midi Purification Kit. Restriction enzymes, ligases and other DNA-manipulating enzymes were used according to their manufacturer’s instructions.

The *aro* genes involving shikimate pathway, i.e., *aroA* (Genbank accession number, NP_390853.1), *aroB* (NP_390151.1), *aroC* (NP_390189.1), and *aroD* (NP_390444.2) were PCR amplified from genomic DNA of *B. subtilis* strain 168 using primers listed in Table 3. The PCR products were digested with *Spe*I and *Xma*I and then cloned into the same digested pHCMC04 [25]. All plasmids (Table 3) constructed in this study were verified by DNA sequencing. Transformation of chemically competent cells of *E. coli* DH5 and of *B. subtilis* were carried out according to the manufacturer’s manual or by the method of Spizizen [26]. In order to simultaneous overexpression of *aroA* and *aroD* in B. subtilis, *aroD* was cloned into the pDG148-Stu vector [27], and the resulted plasmid was named pDGSA*AroD* (Table 3). Expression of the cloned *aro* genes in *B. subtilis* was induced by either 5 g/L of xylose for pHCMC04 derivatives or 0.05 mM IPTG for pDG148-Stu derivative.

Cloning and overexpression of *tkt* (NP_389672.10) in *B. subtilis* were performed with the plasmid pMUTIN4 [28]. The *tkt* with its ribosome binding site (RBS) was amplified by using primers listed in Table 3 and was cloned into the *Bam*HI-*Xma*I treated backbone of pMUTIN4. Transformation of *E. coli* DH5α and *B. subtilis* BSSA/pSA*AroA*/pDGSA*AroD* and selection of transformants were performed as previously described. The *B. subtilis* transformants were selected on LB plates containing 15 ug/mL erythromycin, 5 ug/mL chloramphenicol, 30 ug/mL neomycin and then analyzed by PCR to ensure the integration of a single copy of the plasmid into the target gene on the chromosome.

### Construction of an inducible *pyk* mutant

The plasmid pMUTIN4 was also used to produce knockout mutations [28]. The fragment of 5’ end of *pyk* (NP_390796.1) with its RBS was amplified from *Bacillus subtilis* 168 chromosomal DNA using primers listed in Table 3, and was cloned into the *Bam*HI-*Xma*I treated backbone of pMUTIN4. Transformation of *E. coli* DH5α and *B. subtilis* BSSA/pSA*AroA*/pDGSA*AroD* were performed as previously described. The *B. subtilis* transformants were selected on LB plates containing 15 μg/mL erythromycin, 5 μg/mL chloramphenicol, 30 μg/mL neomycin and 1 mM IPTG and then analyzed by PCR to ensure that the *pyk* gene had been knocked out.

### Growth and cultivation of *B. subtilis* with ^13^C-labeled glucose

The chemically defined M9 minimal medium [29] was applied for the ^13^C labeling experiment. Glucose was added to M9 minimal medium as either entirely the [1-^13^C]-labeled isotope isomer (99%; Cambridge Isotope Laboratories Inc., Andover, MA) or as a mixture of 20% (w/w) [U-^13^C] (99%; Cambridge Isotope Laboratories Inc.) and 80% (w/w) unlabeled glucose. *B. subtilis* strains pre-cultivated overnight in 5 mL of M9 minimal medium supplemented with 5 g/L unlabeled glucose, and the precultures (2 mL) were used as inoculum for ^13^C-labeling experiments. The ^13^C-labeling experiments were performed in a 250-mL flask that contained 50 mL of M9 minimal medium, which was supplemented with either 20:80 (w/w) mixture of [U-^13^C] glucose:unlabeled glucose or 100% [1-^13^C] glucose. Temperature and the shaking rate of the incubator were held constant at 37°C and 200 rpm, respectively. When cells reached exponential phase, aliquots of 0.5 mL was aseptically collected from the culture at intervals of 0.5 h. Measure the OD_600_ on a spectrometer to monitor the growth of the cells. For extracellular metabolite (shikimic acid and 3-dehydroshikimic acid) analysis by HPLC, culture samples were centrifuged for 5 min at 4°C and 20,000 *g* to remove the cells. The specific growth rate (μ**)** was defined as the increase in cell biomass concentration per unit time. The specific glucose consumption rate (q_glu_) was calculated during the exponential growth phase as the differential change in glucose with time (t) normalized to the biomass concentration. The specific SA production rate (q_sa_), and the specific 3-dehydroshikicmic acid production rate (q_dhs_) were calculated during the exponential growth phase as the differential change in SA, and 3-dehydroshikicmic acid with time (t) normalized to the biomass concentration, respectively.

### Preparation of cellular hydrolysate from ^13^C-labeled biomass and measurements of ^13^C-labeled amino acids with gas chromatography and mass spectroscopy (GC-MS)

Preparation of cellular hydrolysate from ^13^C-labeled biomass for the GC-MS measurement followed standard protocols [30]. For GC-MS measurement, the amino acids were derivatized with TBDMS and 1% tert-butyldimethylchlorosilane at 85°C for 60 min. The amounts of biomass used for GC-MS measurement was approximately 10 mg of cellular dry weight. GC-MS was carried out using an Agilent GC-7890A gas chromatograph equipped with an Agilent HP-5MS column (30 m × 0.25 mm × 0.25 mm) that was directly connected to an MS-5975C mass spectrometer (Agilent). The following amino acids were detected: Ala, Asp, Glu, Gly, His, Ile, Leu, Lys, Met, Phe, Pro, Ser, Thr, Tyr, and Val.

### Determination of ^13^C-labeled SA and 3-dehydroshikimic acid with LC-MS

Agilent 1290 UHPLC system equipped with ZORBAX 300SB-C18 Column (2.1 mm × 100 mm × 1.8 μm) and Agilent 6530 Q-TOF mass spectrometer fitted with an electrospray ionization (ESI) source were used to measure ^13^C-labeling states of SA and 3-dehydroshikimic acid. SA and 3-dehydroshikimic acid was eluted with mixture of 0.1% formic acid in water (solution C) and acetonitrile (solution D) at ratio of 98:2 (solution C: solution D) and at a flow rate of 0.15 mL/min. MS data was collected in negative ion mode. The capillary voltage was set to 3500 kV, and the fragmentor voltage at 90 V. The drying gas temperature was maintained at 350°C with a flow rate of 11 L/min and a nebulizer pressure of 40 psi.

### Metabolic net-flux analysis

^13^C MFA was a well-established method, and details concerning the general modeling framework of classical stationary ^13^C labeling experiments could be found elsewhere [30]. In the case of U-^13^C labeling, several flux ratios, including (1) Pyr originating from malate, (2) PEP from OAA, (3) OAA from PEP, (4) PEP from transketolase, (5) PEP from the PP pathway, (6) pentose-5-phosphate from Glu-6-P, (7) OAA from the glyoxylate shunt, (8) erythrose-4-phosphate from pentose-5-phosphate, (9) Ser from Gly, (10) Gly from Ser, and (11) PEP and E4P from shikimic acid were calculated from the *MDV*_M_ of precursors (Additional file 1: Table S2) with probabilistic equations. The calculating procedures were based on the general principle of flux ratio analysis [30-32] and were performed using a home-made Matlab program. Positional labeling pattern was also analyzed from cells grown exclusively on [1-^13^C] glucose to obtain precise information about the in vivo activities of the PPP and EMP pathways.

For quantification of carbon fluxes in *B. subtilis*, a bioreaction network was constructed. This network includes the reactions of the EMP, PP, and shikimate pathways, as well as the tricarboxylic acid (TCA) cycle and the glyoxylate shunt. The reactions catalyzed by PEP carboxykinase (*v*_10_), pyruvate carboxylase (*v*_11_), and malic enzyme (*v*_12_) were also included. The networks of active pathways identified by flux ratio analysis were used for flux quantification. The following enzyme reactions were considered reversible: phosphoglucose isomerase (*v*_2_), transketolase (*v*_5_ and *v*_7_), transaldolase (*v*_6_), the sequence of glycolytic reactions leading from triose 3-phosphate to PEP (*v*_8_) and malate dehydrogenase (*v*_17_). The metabolic reactions considered as shown in the Additional file 1: Appendix S1.

All the above data, including (1) the stoichiometric reaction matrix, (2) the flux ratios derived from metabolic flux ratio analysis (Additional file 1: Table S3), (3) physiological data, and (4) precursor requirements for biomass synthesis, were utilized to calculate absolute fluxes in millimoles per gram of biomass per hour. Net fluxes were then estimated with Matlab by solving the stoichiometric matrix. The resulting flux distribution was the best fit to available data from both metabolite balances and tracer experiment-based flux ratios within the specified metabolic model.

### Statistical analysis

Monte Carlo approach [33] was applied for statistical analysis.

## Competing interests

The authors declare that they have no competing interests.

## Authors’ contributions

SJL and CY conceived and designed the experiments. DFL and GMA performed the experiments. DFL, YML, QXZ, LXL, BZ, CL, CYJ, CY and SJL analyzed the data. DFL and SJL wrote the manuscript. All authors read and approved the final manuscript.

## Supplementary Material

Additional file 1: Table S1 Mass isotopomer distribution of TBDMS-derivatized protein-bound amino acids and shikimic acid (corrected). **Table S2.** Mass isotopomer distribution of precursors. **Table S3.** Flux ratio of *Bacillus subtilis* BSSA/pHCMC04/pDG148-stu and *Bacillus subtilis* BSSA/pSA*AroA*/pDGSA*AroD*. **Figure S1.**^13^C fractional labeling (FL) of 15 proteinogenic amino acids and shikimic acid from *Bacillus subtilis* BSSA/pHCMC04/pDG148-stu (a) and *Bacillus subtilis* BSSA BSSA/pSA*AroA*/pDGSA*AroD* (b) during exponential growth phase. **Appendix S1.** Stoichiometric reactions implemented in the central metabolic network of *Bacillus subtilis*. Click here for file
