# Supplementary material for: Metabolic flux responses to genetic modification for shikimic acid production by Bacillus subtilis strains
Source: Microb Cell Fact. 2014 Mar 14;13:40. doi: 10.1186/1475-2859-13-40 (PMC4003833; doi:10.1186/1475-2859-13-40)
Supplement: Additional file 1: Table S1 — Mass isotopomer distribution of TBDMS-derivatized protein-bound amino acids and shikimic acid (corrected). Table S2. Mass isotopomer distribution of precursors. Table S3. Flux ratio of Bacillus subtilis BSSA/pHCMC04/pDG148-stu and Bacillus subtilis BSSA/pSAAroA/pDGSAAroD. Figure S1.13C fractional labeling (FL) of 15 proteinogenic amino acids and shikimic acid from Bacillus subtilis BSSA/pHCMC04/pDG148-stu (a) and Bacillus subtilis BSSA BSSA/pSAAroA/pDGSAAroD (b) during exponential growth phase. Appendix S1. Stoichiometric reactions implemented in the central metabolic network of Bacillus subtilis. [file 1475-2859-13-40-S1.doc]

**Additional file 1**

**Table S1：Mass isotopomer distribution of precursors.**

| **Precursors** | ***Bacillus subtilis* BSSA474a** | | | | | | ***Bacillus subtilis* BSSA47407** | | | | | |
| --- | --- | --- | --- | --- | --- | --- | --- | --- | --- | --- | --- | --- |
|  | **m** | **m+1** | **m+2** | **m+3** | **m+4** | **m+5** | **m** | **m+1** | **m+2** | **m+3** | **m+4** | **m+5** |
| ACoA1_2 | 0.808 | 3.31E-11 | 0.192 | 0.000 | 0.000 | 0.000 | 0.794 | 4.67E-12 | 0.206 | 0.000 | 0.000 | 0.000 |
| AKG1_5 | 0.490 | 0.192 | 0.214 | 0.075 | 0.023 | 0.007 | 0.490 | 0.187 | 0.221 | 0.073 | 0.023 | 0.006 |
| AKG2_5 | 0.558 | 0.182 | 0.205 | 0.038 | 0.016 | 0.000 | 0.552 | 0.186 | 0.203 | 0.041 | 0.018 | 0.000 |
| AKG1_2 | 0.738 | 0.191 | 0.071 | 0.000 | 0.000 | 0.000 | 0.713 | 0.188 | 0.099 | 0.000 | 0.000 | 0.000 |
| E4P1_4 | 0.750 | 0.069 | 0.006 | 0.059 | 0.117 | 0.000 | 0.739 | 0.063 | 1.63E-05 | 0.118 | 0.080 | 0.000 |
| Gly1_2 | 0.787 | 0.061 | 0.152 | 0.000 | 0.000 | 0.000 | 0.784 | 0.051 | 0.165 | 0.000 | 0.000 | 0.000 |
| OAA1_4 | 0.588 | 0.185 | 0.122 | 0.080 | 0.024 | 0.000 | 0.577 | 0.192 | 0.125 | 0.082 | 0.024 | 0.000 |
| OAA2_4 | 0.638 | 0.197 | 0.128 | 0.037 | 0.000 | 0.000 | 0.619 | 0.214 | 0.129 | 0.038 | 0.000 | 0.000 |
| OAA1_2 | 0.753 | 0.117 | 0.130 | 0.000 | 0.000 | 0.000 | 0.738 | 0.121 | 0.141 | 0.000 | 0.000 | 0.000 |
| P5P1_5 | 0.693 | 0.047 | 0.092 | 0.068 | 0.016 | 0.084 | 0.676 | 0.095 | 0.049 | 0.071 | 0.000394 | 0.108 |
| P5P1_4 | 0.657 | 0.040 | 0.136 | 0.036 | 0.131 | 0.000 | 0.653 | 0.075 | 0.082 | 0.088 | 0.101 | 0.000 |
| P5P4_5 | 0.780 | 0.099 | 0.121 | 0.000 | 0.000 | 0.000 | 0.760 | 0.028 | 0.213 | 0.000 | 0.000 | 0.000 |
| PEP1_3 | 0.638 | 0.314 | 0.048 | 0.000 | 0.000 | 0.000 | 0.764 | 0.060 | 0.020 | 0.157 | 0.000 | 0.000 |
| PYR1_3 | 0.755 | 0.057 | 0.034 | 0.154 | 0.000 | 0.000 | 0.755 | 0.058 | 0.034 | 0.154 | 0.000 | 0.000 |
| PYR2_3 | 0.771 | 0.046 | 0.183 | 0.000 | 0.000 | 0.000 | 0.779 | 0.040 | 0.181 | 0.000 | 0.000 | 0.000 |
| PYR1_2 | 0.764 | 0.068 | 0.168 | 0.000 | 0.000 | 0.000 | 0.741 | 0.080 | 0.179 | 0.000 | 0.000 | 0.000 |
| Ser1_3 | 0.755 | 0.056 | 0.038 | 0.150 | 0.000 | 0.000 | 0.736 | 0.062 | 0.034 | 0.168 | 0.000 | 0.000 |
| Ser2_3 | 0.789 | 0.035 | 0.176 | 0.000 | 0.000 | 0.000 | 0.765 | 0.052 | 0.184 | 0.000 | 0.000 | 0.000 |
| Ser1_2 | 0.759 | 0.062 | 0.178 | 0.000 | 0.000 | 0.000 | 0.769 | 0.067 | 0.164 | 0.000 | 0.000 | 0.000 |
| OAA2_3 | 0.722 | 0.193 | 0.086 | 0.000 | 0.000 | 0.000 | 0.719 | 0.192 | 0.089 | 0.000 | 0.000 | 0.000 |

**Table S2：** **Mass isotopomer distribution of TBDMS-derivatized protein-bound amino acids and shikimic acid (corrected).**

The symbols of fragments denoted the cracking patterns of TBDMS-derivatized amino acids: (M-15)^+^, (M-57)^+^, (M-85)^+^, (M-159)^+^, f302, and side chain (sc) fragments. M-sa; shikimic acid.

| **Fragments** | ***Bacillus subtilis* BSSA474a** | | | | | | | | | | ***Bacillus subtilis* BSSA47407** | | | | | | | | | |
| --- | --- | --- | --- | --- | --- | --- | --- | --- | --- | --- | --- | --- | --- | --- | --- | --- | --- | --- | --- | --- |
|  | **m** | **m+1** | **m+2** | **m+3** | **m+4** | **m+5** | **m+6** | **m+7** | **m+8** | **m+9** | **m** | **m+1** | **m+2** | **m+3** | **m+4** | **m+5** | **m+6** | **m+7** | **m+8** | **m+9** |
| M-ala-057 | 0.755 | 0.057 | 0.034 | 0.154 | 0.000 | 0.000 | 0.000 | 0.000 | 0.000 | 0.000 | 0.755 | 0.057 | 0.034 | 0.154 | 0.000 | 0.000 | 0.000 | 0.000 | 0.000 | 0.000 |
| M-ala-085 | 0.771 | 0.046 | 0.183 | 0.000 | 0.000 | 0.000 | 0.000 | 0.000 | 0.000 | 0.000 | 0.779 | 0.040 | 0.181 | 0.000 | 0.000 | 0.000 | 0.000 | 0.000 | 0.000 | 0.000 |
| M-asx-057 | 0.593 | 0.180 | 0.124 | 0.080 | 0.023 | 0.000 | 0.000 | 0.000 | 0.000 | 0.000 | 0.571 | 0.197 | 0.123 | 0.086 | 0.023 | 0.000 | 0.000 | 0.000 | 0.000 | 0.000 |
| M-asx-085 | 0.648 | 0.191 | 0.122 | 0.038 | 0.000 | 0.000 | 0.000 | 0.000 | 0.000 | 0.000 | 0.621 | 0.211 | 0.128 | 0.040 | 0.000 | 0.000 | 0.000 | 0.000 | 0.000 | 0.000 |
| M-asx-302 | 0.753 | 0.117 | 0.130 | 0.000 | 0.000 | 0.000 | 0.000 | 0.000 | 0.000 | 0.000 | 0.738 | 0.121 | 0.141 | 0.000 | 0.000 | 0.000 | 0.000 | 0.000 | 0.000 | 0.000 |
| M-glx-057 | 0.490 | 0.192 | 0.214 | 0.075 | 0.023 | 0.007 | 0.000 | 0.000 | 0.000 | 0.000 | 0.490 | 0.187 | 0.221 | 0.073 | 0.023 | 0.006 | 0.000 | 0.000 | 0.000 | 0.000 |
| M-glx-085 | 0.556 | 0.181 | 0.207 | 0.041 | 0.015 | 0.000 | 0.000 | 0.000 | 0.000 | 0.000 | 0.559 | 0.176 | 0.205 | 0.043 | 0.017 | 0.000 | 0.000 | 0.000 | 0.000 | 0.000 |
| M-glx-302 | 0.738 | 0.191 | 0.071 | 0.000 | 0.000 | 0.000 | 0.000 | 0.000 | 0.000 | 0.000 | 0.713 | 0.188 | 0.099 | 0.000 | 0.000 | 0.000 | 0.000 | 0.000 | 0.000 | 0.000 |
| M-gly-057 | 0.787 | 0.061 | 0.152 | 0.000 | 0.000 | 0.000 | 0.000 | 0.000 | 0.000 | 0.000 | 0.784 | 0.051 | 0.165 | 0.000 | 0.000 | 0.000 | 0.000 | 0.000 | 0.000 | 0.000 |
| M-gly-085 | 0.820 | 0.180 | 0.000 | 0.000 | 0.000 | 0.000 | 0.000 | 0.000 | 0.000 | 0.000 | 0.804 | 0.196 | 0.000 | 0.000 | 0.000 | 0.000 | 0.000 | 0.000 | 0.000 | 0.000 |
| M-his-057 | 0.561 | 0.169 | 0.083 | 0.072 | 0.026 | 0.070 | 0.018 | 0.000 | 0.000 | 0.000 | 0.543 | 0.208 | 0.057 | 0.066 | 0.012 | 0.086 | 0.029 | 0.000 | 0.000 | 0.000 |
| M-his-159 | 0.531 | 0.156 | 0.117 | 0.054 | 0.112 | 0.030 | 0.000 | 0.000 | 0.000 | 0.000 | 0.529 | 0.192 | 0.084 | 0.091 | 0.103 | 0.000 | 0.000 | 0.000 | 0.000 | 0.000 |
| M-his-302 | 0.780 | 0.099 | 0.121 | 0.000 | 0.000 | 0.000 | 0.000 | 0.000 | 0.000 | 0.000 | 0.760 | 0.028 | 0.213 | 0.000 | 0.000 | 0.000 | 0.000 | 0.000 | 0.000 | 0.000 |
| M-ile-015 | 0.469 | 0.170 | 0.219 | 0.101 | 0.041 | 0.000 | 0.000 | 0.000 | 0.000 | 0.000 | 0.465 | 0.168 | 0.258 | 0.051 | 0.056 | 0.001 | 0.000 | 0.000 | 0.000 | 0.000 |
| M-ile-085 | 0.502 | 0.181 | 0.217 | 0.069 | 0.025 | 0.006 | 0.000 | 0.000 | 0.000 | 0.000 | 0.492 | 0.187 | 0.221 | 0.068 | 0.025 | 0.006 | 0.000 | 0.000 | 0.000 | 0.000 |
| M-leu-015 | 0.505 | 0.084 | 0.284 | 0.067 | 0.052 | 0.008 | 0.000 | 0.000 | 0.000 | 0.000 | 0.503 | 0.074 | 0.334 | 0.039 | 0.049 | 0.000 | 0.000 | 0.000 | 0.000 | 0.000 |
| M-leu-085 | 0.504 | 0.168 | 0.228 | 0.066 | 0.027 | 0.006 | 0.000 | 0.000 | 0.000 | 0.000 | 0.498 | 0.169 | 0.234 | 0.063 | 0.030 | 0.006 | 0.000 | 0.000 | 0.000 | 0.000 |
| M-lys-057 | 0.467 | 0.183 | 0.140 | 0.133 | 0.045 | 0.023 | 0.009 | 0.000 | 0.000 | 0.000 | 0.467 | 0.189 | 0.158 | 0.119 | 0.033 | 0.022 | 0.011 | 0.000 | 0.000 | 0.000 |
| M-lys-159 | 0.487 | 0.202 | 0.215 | 0.060 | 0.030 | 0.005 | 0.000 | 0.000 | 0.000 | 0.000 | 0.486 | 0.201 | 0.214 | 0.073 | 0.020 | 0.005 | 0.000 | 0.000 | 0.000 | 0.000 |
| M-lys-302 | 0.637 | 0.278 | 0.085 | 0.000 | 0.000 | 0.000 | 0.000 | 0.000 | 0.000 | 0.000 | 0.640 | 0.268 | 0.091 | 0.000 | 0.000 | 0.000 | 0.000 | 0.000 | 0.000 | 0.000 |
| M-met-057 | 0.504 | 0.236 | 0.126 | 0.100 | 0.033 | 0.001 | 0.000 | 0.000 | 0.000 | 0.000 | 0.444 | 0.263 | 0.139 | 0.091 | 0.062 | 0.000 | 0.000 | 0.000 | 0.000 | 0.000 |
| M-met-085 | 0.528 | 0.261 | 0.130 | 0.073 | 0.008 | 0.000 | 0.000 | 0.000 | 0.000 | 0.000 | 0.523 | 0.283 | 0.129 | 0.048 | 0.016 | 0.000 | 0.000 | 0.000 | 0.000 | 0.000 |
| M-phe-057 | 0.902 | 0.096 | 0.000 | 0.001 | 0.000 | 0.000 | 0.000 | 0.000 | 0.000 | 0.000 | 0.897 | 0.099 | 0.004 | 0.000 | 0.000 | 0.000 | 0.000 | 0.000 | 0.000 | 0.000 |
| M-phe-085 | 0.888 | 0.085 | 0.002 | 0.001 | 0.002 | 0.016 | 0.004 | 0.001 | 0.000 | 0.000 | 0.899 | 0.075 | 0.003 | 0.000 | 0.001 | 0.015 | 0.004 | 0.001 | 0.000 | 0.000 |
| M-phe-302 | 0.990 | 0.010 | 0.000 | 0.000 | 0.000 | 0.000 | 0.000 | 0.000 | 0.000 | 0.000 | 0.976 | 0.024 | 0.000 | 0.000 | 0.000 | 0.000 | 0.000 | 0.000 | 0.000 | 0.000 |
| M-phe-sc | 0.774 | 0.091 | 0.034 | 0.005 | 0.016 | 0.036 | 0.026 | 0.018 | 0.000 | 0.000 | 0.765 | 0.091 | 0.033 | 0.000 | 0.018 | 0.046 | 0.031 | 0.017 | 0.000 | 0.000 |
| M-pro-057 | 0.626 | 0.153 | 0.144 | 0.051 | 0.021 | 0.005 | 0.000 | 0.000 | 0.000 | 0.000 | 0.635 | 0.164 | 0.132 | 0.048 | 0.018 | 0.003 | 0.000 | 0.000 | 0.000 | 0.000 |
| M-pro-085 | 0.561 | 0.182 | 0.204 | 0.036 | 0.017 | 0.000 | 0.000 | 0.000 | 0.000 | 0.000 | 0.545 | 0.196 | 0.200 | 0.039 | 0.019 | 0.000 | 0.000 | 0.000 | 0.000 | 0.000 |
| M-ser-057 | 0.755 | 0.056 | 0.038 | 0.150 | 0.000 | 0.000 | 0.000 | 0.000 | 0.000 | 0.000 | 0.736 | 0.062 | 0.034 | 0.168 | 0.000 | 0.000 | 0.000 | 0.000 | 0.000 | 0.000 |
| M-ser-085 | 0.789 | 0.035 | 0.176 | 0.000 | 0.000 | 0.000 | 0.000 | 0.000 | 0.000 | 0.000 | 0.765 | 0.052 | 0.184 | 0.000 | 0.000 | 0.000 | 0.000 | 0.000 | 0.000 | 0.000 |
| M-ser-302 | 0.759 | 0.062 | 0.178 | 0.000 | 0.000 | 0.000 | 0.000 | 0.000 | 0.000 | 0.000 | 0.769 | 0.067 | 0.164 | 0.000 | 0.000 | 0.000 | 0.000 | 0.000 | 0.000 | 0.000 |
| M-thr-057 | 0.584 | 0.189 | 0.121 | 0.080 | 0.026 | 0.000 | 0.000 | 0.000 | 0.000 | 0.000 | 0.584 | 0.186 | 0.127 | 0.079 | 0.024 | 0.000 | 0.000 | 0.000 | 0.000 | 0.000 |
| M-thr-085 | 0.627 | 0.204 | 0.133 | 0.036 | 0.000 | 0.000 | 0.000 | 0.000 | 0.000 | 0.000 | 0.617 | 0.216 | 0.131 | 0.036 | 0.000 | 0.000 | 0.000 | 0.000 | 0.000 | 0.000 |
| M-thr-sc | 0.614 | 0.271 | 0.114 | 0.000 | 0.000 | 0.000 | 0.000 | 0.000 | 0.000 | 0.000 | 0.622 | 0.262 | 0.116 | 0.000 | 0.000 | 0.000 | 0.000 | 0.000 | 0.000 | 0.000 |
| M-val-057 | 0.585 | 0.087 | 0.151 | 0.140 | 0.010 | 0.026 | 0.000 | 0.000 | 0.000 | 0.000 | 0.584 | 0.076 | 0.155 | 0.142 | 0.011 | 0.032 | 0.000 | 0.000 | 0.000 | 0.000 |
| M-val-085 | 0.612 | 0.069 | 0.268 | 0.021 | 0.030 | 0.000 | 0.000 | 0.000 | 0.000 | 0.000 | 0.601 | 0.067 | 0.283 | 0.016 | 0.032 | 0.000 | 0.000 | 0.000 | 0.000 | 0.000 |
| M-val-302 | 0.764 | 0.068 | 0.168 | 0.000 | 0.000 | 0.000 | 0.000 | 0.000 | 0.000 | 0.000 | 0.741 | 0.080 | 0.179 | 0.000 | 0.000 | 0.000 | 0.000 | 0.000 | 0.000 | 0.000 |
| M-tyr-057 | 0.916 | 0.083 | 0.000 | 0.001 | 0.000 | 0.000 | 0.000 | 0.000 | 0.000 | 0.000 | 0.928 | 0.066 | 0.003 | 0.002 | 0.000 | 0.000 | 0.000 | 0.000 | 0.000 | 0.000 |
| M-tyr-085 | 0.906 | 0.072 | 0.002 | 0.003 | 0.005 | 0.007 | 0.000 | 0.002 | 0.002 | 0.000 | 0.720 | 0.060 | 0.012 | 0.001 | 0.000 | 0.003 | 0.005 | 0.172 | 0.026 | 0.000 |
| M-tyr-302 | 0.981 | 0.019 | 0.000 | 0.000 | 0.000 | 0.000 | 0.000 | 0.000 | 0.000 | 0.000 | 0.976 | 0.022 | 0.000 | 0.000 | 0.000 | 0.000 | 0.000 | 0.000 | 0.000 | 0.000 |
| M-sa | 0.567 | 0.104 | 0.030 | 0.156 | 0.102 | 0.010 | 0.012 | 0.018 | 0.000 | 0.000 | 0.574 | 0.097 | 0.029 | 0.156 | 0.107 | 0.008 | 0.011 | 0.018 | 0.000 | 0.000 |

**Table S3：Flux ratio of *B. subtilis* BSSA/pHCMC04/pDG148-stu and BSSA/pSA*AroA*/pDGSA*AroD*. In certain cases, the MS data permits the determination only of upper bounds (ub) or lower bounds (lb) on the origin of intermediates. E4P, Erythrose-4-phosphate; G6P, Glc-6-P; GOX, glyoxylate shunt; Mal, malate; P5P, pentose-5-phosphate; tkt, transketolase.**

|  | *Bacillus subtilis* BSSA/pHCMC04/pDG148-stu | *Bacillus subtilis* BSSA/pSA*AroA*/ pDGSA*AroD* |
| --- | --- | --- |
| f_3PG_from_EMP | 0.718±0.010 | 0.764±0.007 |
| f_PEP_from_OAA(ub) | 0.429±0.011 | 0.121±0.003 |
| f_PEP_from_OAA(lb) | 0.038±0.001 | 0.043±0.002 |
| f_Pyr_from_Mal(ub) | 0 | 0 |
| f_Pyr_from_Mal(lb) | 0 | 0 |
| f_OAA_from_PYR | 0.488±0.006 | 0.471±0.003 |
| f_3PG_from_tkt | 0.155±0.004 | 0.168±0.001 |
| f_3PG_from_PP | 0.388±0.007 | 0.420±0.005 |
| f_P5P_from_G6P | 0.392±0.011 | 0.448±0.013 |
| f_P5P_from_E4P | 0.076±0.007 | 0.234±0.011 |
| l_CO2 | 0.184±0.005 | 0.199±0.006 |
| GOX_indicator | 0 | 0 |


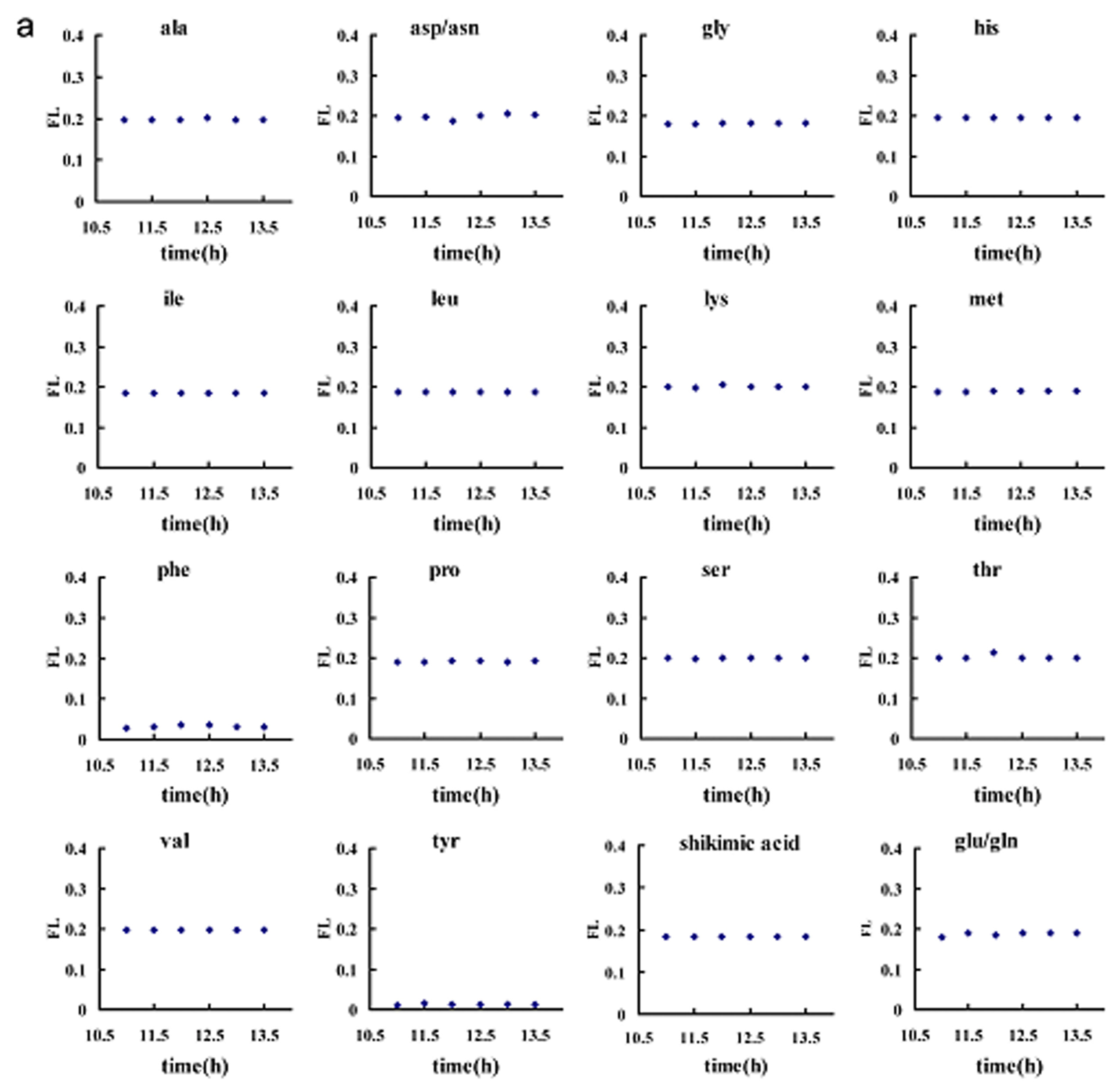


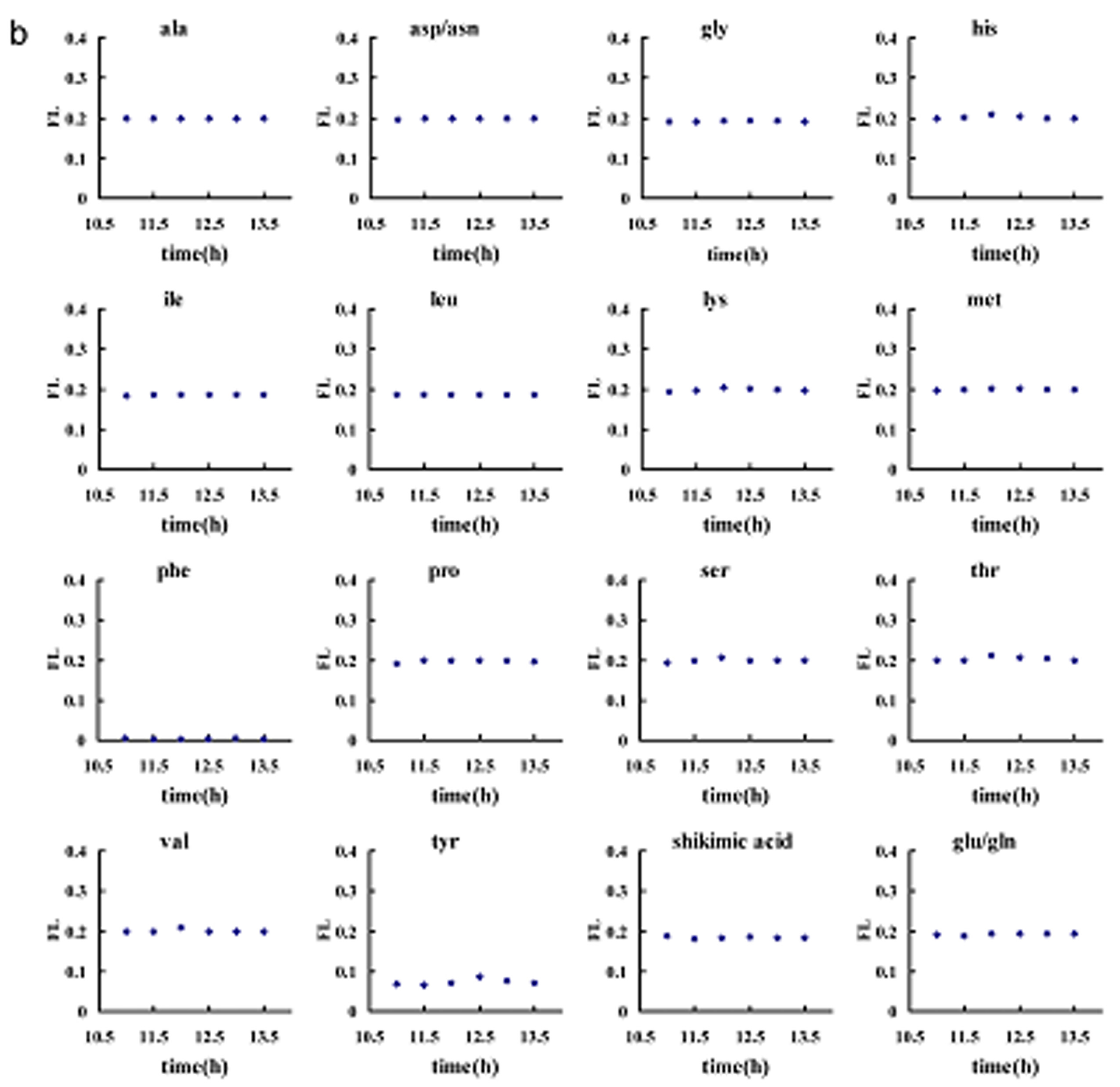


**Figure S1** **^13^C fractional labeling (FL) of 15 proteinogenic amino acids and shikimic acid from *B. subtilis*** BSSA/pHCMC04/pDG148-stu **(a) and** BSSA/pSA*AroA*/pDGSA*AroD* **(b) during exponential growth phase.** The FL data were obtained in experiments with 20% [U-^13^C] and 80% unlabeled glucose. Amino acids were denoted with their three-letter abbreviations. The points indicated the times of biomass sampling for ^13^C labeling pattern analysis. FL was calculated on the basis of GC-MS data which had been corrected for the natural isotope abundance.

**APPENDIX S1**

The central metabolic network in *Bacillus subtilis* with the stoichiometric reactions was given as follows.

| **NO.** | **Reaction** | **Enzymes** |
| --- | --- | --- |
| *v*_1_ | GLU + ATP → G6P | EC:2.7.1.2(NP_390365.2) |
| *v*_2_ | G6P = F6P | EC:5.3.1.9(NP_391013.2) |
| *v*_3_ | F6P + ATP = 2*GAP | EC:2.7.1.11(NP_390797.1)  EC:4.1.2.13(NP_391593.1) |
| *v*_4_ | G6P → P5P + CO_2_ + 2NADPH | EC:1.1.1.49(NP_390266.2)  EC:3.1.1.31(NP_389184.1)  EC:1.1.1.44(NP_390267.2)  EC:5.1.3.1(NP_389461.1) |
| *v*_5_ | 2*P5P = S7P + GAP | EC:2.2.1.1(NP_389672.1) |
| *v*_6_ | S7P + T3P = E4P + F6P | EC:2.2.1.2(NP_391592.3) |
| *v*_7_ | P5P + E4P = F6P + GAP | EC:2.2.1.1(NP_389672.1) |
| *v*_8_ | GAP = PEP + ATP + NADH | EC:1.2.1.12(NP_390780.1)  EC:2.7.2.3(NP_391273.1)  EC:5.4.2.1(NP_391271.1)  EC:4.2.1.11(NP_391270.1) |
| *v*_9_ | PEP → PYR + ATP | EC:2.7.1.40(NP_390796.1) |
| *v*_10_ | OAA→ PEP + CO_2_+ ATP | EC:4.1.1.49(NP_390934.2) |
| *v*_11_ | PYR + CO_2_+ ATP→OAA | EC:6.4.1.1(NP_389369.1) |
| *v*_12_ | MAL → PYR + CO_2_ + NADPH | EC:1.1.1.40(NP_390800.1)  EC:1.1.1.38(NP_390236.1)  EC:1.1.1.38(NP_390866.1) |
| *v*_13_ | PYR → AcCoA + CO_2_ + NADH | EC:1.2.4.1(NP_388687.1)  EC:2.3.1.12(NP_388689.1) |
| *v*_14_ | OAA + AcCoA → ICT | EC:2.3.3.1(NP_388825.2)  EC:4.2.1.3(NP_389683.1) |
| *v*_15_ | ICT → AKG + CO_2_ + NADPH | EC:1.1.1.41(NP_390791.1) |
| *v*_16_ | AKG →MAL + CO2 + 1.5×ATP + 2×NADH | EC:1.2.4.2(NP_389819.3)  EC:2.3.1.61(NP_389818.2)  EC:6.2.1.5(NP_389491.1)  EC:1.3.99.1(NP_390721.1)  EC:4.2.1.2(NP_391184.1) |
| *v*_17_ | MAL = OAA + NADH | EC:1.1.1.37(NP_390790.1) |
| *v*_18_ | biomass synthesis |  |
| *v*_19_ | PEP + E4P+ NADPH → SA+2Pi | EC:2.5.1.54(NP_390853.1)  EC:4.2.3.4(NP_390151.1)  EC:4.2.1.10(NP_390189.1)  EC:1.1.1.25(NP_390444.2) |
| *v*_20_ | PEP + E4P → DHS+2Pi | EC:2.5.1.54(NP_390853.1)  EC:4.2.3.4(NP_390151.1)  EC:4.2.1.10(NP_390189.1) |

**Abbreviations :**

**G6P** glucose-6-phosphate

**P5P** pentose-5-phosphate

**F6P** fructose-6-phosphate

**GAP** glyceraldehyde 3-phosphate

**Pyr** pyruvate

**S7P** sedoheptulose-7-phosphate

**E4P** erythrose-4-phosphate

**PEP** phosphoenolpyruvate

**AcCoA** acetyl coenzyme A

**ICT** isocitrate

**AKG** α-ketoglutarate

**OAA** oxaloacetate

**Mal** malate

**DHS** 3-dehydroshikimic acid

**SA** shikimic acid

**Pi** phosphate
